# New literacy challenge for the twenty-first century: genetic knowledge is poor even among well educated

**DOI:** 10.1007/s12687-018-0363-7

**Published:** 2018-03-28

**Authors:** Robert Chapman, Maxim Likhanov, Fatos Selita, Ilya Zakharov, Emily Smith-Woolley, Yulia Kovas

**Affiliations:** 1 Department of Psychology, Goldsmiths, University of London, New Cross, London, UK; 2 Tomsk State University, Tomsk, Russia; 3 IBL Consultancy, London, UK; 4 Psychological Institute of the Russian Academy of Education, Moscow, Russia; 5 Social, Genetic and Developmental Psychiatry Centre, King’s College, London, UK

**Keywords:** Genetic knowledge, Genetic literacy, Health, Genetic testing, Demographic differences

## Abstract

We live in an age of rapidly advancing genetic research. This research is generating new knowledge that has implications for personal health and well-being. The present study assessed the level of genetic knowledge and personal engagement with genetics in a large sample (*N* = 5404) of participants. Participants received secondary education in 78 countries, with the largest samples from Russia, the UK and the USA. The results showed significant group differences in genetic knowledge between different countries, professions, education levels and religious affiliations. Overall, genetic knowledge was poor. The questions were designed to assess basic genetic literacy. However, only 1.2% of participants answered all 18 questions correctly, and the average score was 65.5%. Genetic knowledge was related to peoples’ attitudes towards genetics. For example, those with greater genetic knowledge were on average more willing to use genetic knowledge for their personal health management. Based on the results, the paper proposes a number of immediate steps that societies can implement to empower the public to benefit from ever-advancing genetic knowledge.

## Introduction

Literacy, the ability to read and write is arguably the most valuable tool an individual has. Society’s efforts to equip all citizens with literacy have paid off in terms of progress and improved quality of life (McCracken et al. 2009). The rapid advancements in genetic science in the twenty-first century have brought about the need for a new kind of literacy—genetic literacy. This involves knowing the basic units of the genome and the rules through which they assemble into meaningful patterns as well as understanding the ways through which vast amounts of information can be generated from simple genomic elements. In the genomic era, understanding genetic information is becoming increasingly crucial for all aspects of our lives, including health (Dudlicek et al. 2004) and education (Asbury and Plomin 2013).

There are at least five reasons why genetic literacy is crucial. First, a wealth of findings leaves us with little doubt that practically all human traits are influenced by genes (Collins 2010; Plomin et al. 2016). Second, the cost and time taken to genotype the entire DNA sequence of an individual are continuously reducing (Goyal et al. 2017). Third, many developed countries have already made genetic screening of newborns mandatory—for the detection and early treatment/intervention for several genetic conditions (Pourfarzam and Zadhoush 2013). In the future, this genotyping of a few genetic variants could be supplemented with whole-genome sequencing conducted soon after birth (Berg et al. 2017). Fourth, genetic discoveries continuously push the boundaries of our imagination: what seems impossible one day, quickly becomes a routine. Today, we can already edit the genome (Barrangou et al. 2007) with gene-editing technologies advancing everyday (Gaudelli et al. 2017). Soon, gene editing may be used as a treatment for diseases, such as cancer (Cyranoski 2016; Reardon 2016) or sight loss (Heier et al. 2017). Prediction of behavioural outcomes from DNA information is also becoming increasingly more precise (Selzam et al. 2017). Fifth, genetic advancements reveal fundamental and unique information on each human that has long-term consequences, not only for the individual, but also for family members (Dyer 2015). Once sequenced, the genome of a person provides progressively more information, as new genetic discoveries are made.

Therefore, although all sciences are important, genetic science is crucial as it touches all aspects of human existence. A person’s DNA sequence reveals information about the past, the present and the future; has implications for fundamental human rights, such as privacy (Lowrance and Collins 2007); and provides an essential tool that can serve many purposes in everyday life, including medicine (e.g. Archibald et al. 2017; Rana et al. 2017; Zhou et al. 2017) and forensics (Roewer 2013). Universal genetic literacy will empower people to make informed decisions on the use of their genetic information. In addition, genetically literate societies will be better equipped to develop policies for ethical and fair distribution of benefits resulting from genetic science. Genetic literacy is not just about raising knowledge. It is also about enabling people to express informed opinions and engage in discussions and debates regarding applications of genetic knowledge. These include gene editing, prenatal screening, and preventative intervention based on probabilistic information.

The first step towards achieving universal genetic literacy is to evaluate people’s genetic knowledge and attitudes towards genetics. Although there have been several studies looking at genetic literacy, these have focused on medical genetics, biology and evolution (Carver et al. 2017), and mostly explored undergraduate populations (Bowling et al. 2008; Carver et al. 2017). The results of these studies suggest that genetic knowledge is insufficient in the general population (Lanie et al. 2004) and in non-science undergraduate students (Bowling et al. 2008). For example, only 34% of 62 respondents, recruited through a random digit dialling method in the continental USA, knew that genes are stored in every cell of the body (Lanie et al. 2004).

The present study administered the International Genetic Literacy and Attitudes Survey (iGLAS) to a large sample of participants from diverse demographic backgrounds, stratified for analyses by age, education, occupation, country of residence, secondary education and religious and political affiliations. We measured genetic knowledge (GK) and attitudes towards genetics, including how likely participants were to undergo genetic testing, the importance of genetic research in education and how genetic information should be used in legal cases.

Based on previous research, we formulated the following ten hypotheses:

### Genetic knowledge (GK)

- Hypothesis 1: Average GK, as evaluated by iGLAS, will be poor.

Previous research has identified that 85% of states in the USA had inadequate standards for genetics education (Dougherty et al. 2011). Another qualitative study found limited genetic literacy in a rural Mexican-American population in relation to family risk of disease (Malen et al. 2016). In contrast, one study (Schmidlen et al. 2016) concluded that their respondents had ‘good’ genetic knowledge related to medicine. However, their sampling was restricted as participants were generally older (mean age 50), Caucasian (90%) and affluent (49% of participants had a household income over $US 100,000).

- Hypothesis 2: Participants’ estimates of heritability for different traits will be under- or over-estimated, mirroring misconceptions about control over traits.

A wealth of findings demonstrates that intuitive views on heritability (the proportion of variance in a trait in a population due to inherited genetic factors) are often wrong (Kovas et al. 2016). Errors that people make in heritability estimates are unlikely to be random. Based on much experience of public engagement events, we expect that participants will underestimate heritability of traits which are seen to be under conscious control and more changeable (e.g. weight, motivation, achievement). In contrast, they will overestimate heritability of traits, which are often considered more fixed (e.g. eye colour, height, IQ).

### GK by demographic characteristics

- Hypothesis 3: Average GK will differ across countries.

Although problems with genetics education have been identified in a number of countries (Dougherty et al. 2011; Challen et al. 2005), there are cross-country differences in secondary education curricula and policies. There are also differences across countries in relevant legislative provisions and in media coverage of genetic findings.

- Hypothesis 4: Levels of GK will vary as a function of an individual’s profession/occupation.

Previous studies showed that representatives of some professions, such as nursing (Calzone et al. 2010), are able to implement genetics knowledge to improve their daily practice. Out of the five occupations considered in this study (doctors, lawyers, teachers, university lecturers and office workers), we expect doctors to be the most genetically literate.

- Hypothesis 5: Parents will on average have higher GK than people who don’t have children.

Parents are usually active in seeking information about child development and thus may acquire more genetic knowledge. In addition, parents who have more than one child and observe significant differences between their children may become more aware of the complexity of gene/environment processes.

- Hypothesis 6: Higher levels of education will be associated with greater GK.

Research has shown that higher general education levels usually correlate with higher scientific literacy (Drummond and Fischhoff 2017; Funk 2017). It can therefore be expected that genetic literacy will also be positively associated with education levels.

- Hypothesis 7: Participants who identify with a religious faith will, on average, show poorer GK than those who do not.

Since the publication of ‘On the Origin of Species by Means of Natural Selection’ (Darwin 1859), there has been contention about the relationship between evolution, genetics and religion (Allum et al. 2014, 2017; Curry 2009). For a full discussion on this topic, see Clark 2014. Previous research has also established a negative link between religiosity and science literacy (Sherkat 2011).

- Hypothesis 8: There will be an average difference in the level of GK between people identifying as politically liberal or conservative.

A recent study identified that conservative people are more likely to purchase literature on applied, commercial sciences (e.g. medicine and climate change), while liberals are more attracted to fundamental science (e.g. physics and zoology) (Shi et al. 2017). Therefore, it could be expected that liberals would have higher levels of GK. However, liberals have also been found to show greater resistance to the consensus over the positive benefits of genetically modified food (Berezow and Campbell 2014). Liberals may also be more likely to reject the notion of genetics playing a role in individual differences, especially in education (‘The Rise and Fall of the Meritocracy - BBC Radio 4’, 2017).

### GK and views on genetics

- Hypothesis 9: Participants with greater GK will consider genetic effects less deterministic.

Based on previous research (Shaw et al. 2008), popular media outlets around the world continue to report genetic findings in binary and deterministic terms, often with misleading headlines—a damaging practice in an era of scrolling news (Condit et al. 2001) (see O’Neill 2015). People holding greater genetic knowledge may be less susceptible to such misinformation.

- Hypothesis 10: Participants with greater GK will on average be more willing to undergo genetic testing.

There has historically been strong resistance to gene testing and therapy in humans (see https://www.gov.uk/government/publications/chief-medical-officer-annual-report-2016-generation-genome for a discussion). However, there is evidence that people are becoming more accepting of genetic testing in certain contexts, presumably with increased relevant knowledge. For example, 85% of 2000 respondents from a Russian urban population expressed positivity towards undergoing predictive genetic testing for preventable health conditions (Makeeva et al. 2010).

## Methods

The International Genetic Literacy and Attitudes Survey (iGLAS) was developed by a collaborative team from Goldsmiths, University of London; Tomsk State University; and The Accessible Genetics Consortium (TAGC). iGLAS consists of four parts: knowledge, attitudes, a personality measure—the shortened Big Five inventory (Rammstedt and John 2007) and a demographic survey. Questions were formatted in several ways to help reduce the effects of common method variance (Lindell and Whitney 2001). The formats of the questions were yes/no, multiple choice, Likert scales (vertical and horizontal presentation), slider scales, dropdowns, radio buttons, checkboxes and free text. A short sample of the English language version of iGLAS that includes all the items analysed in this paper can be found at https://goldpsych.eu.qualtrics.com/jfe/form/SV_9zOfCcGhht7qwy9. Information on the validation of iGLAS can be found in Chapman et al. (in press). The latest version of the study can be found at http://tagc.world/iglas/.

The genetic knowledge (GK) section of iGLAS, the focus of the current paper, consisted of 18 questions. These questions were developed by experts in the fields of genetics, psychology, law, ethics and education to assess a basic, functional, level of genetic literacy. We created a GK score by summing up all correct answers. An abbreviated version of each GK question and percentage correct answers for each question are presented in Table 1, for the total sample and for different demographic groups.

**Table 1:** Percentage of correct answers for each GK question for the total sample and split by demographic groups (with participant numbers in brackets)

Question (shortened/rephrased for this table)	Total sample (5310) (%)	Christian (1093) (%)	Atheist (1349) (%)	Legal Practitioner (90) (%)	Teachers (244) (%)	UG Psychology (112) (%)	Parents (1762) (%)	Men (1919) (%)	Women (3301) (%)
What is a genome?	53	47	58	54	61	55	57	58	50
What 4 letter groups represent the base units of DNA?	76	68	82	54	67	66	71	83	73
In humans, DNA is packaged into how many pairs of chromosomes?	82	76	86	73	84	89	79	84	80
What is the main function of all genes?	99	98	99	100	98	91	99	99	99
What is variable DNA?	57	51	62	43	58	58	56	58	60
On average, how much of their total DNA is the same in two people selected at random?	60	43	76	47	50	35	58	79	49
How many copies of each gene do we have in each cell?	46	40	54	34	49	45	45	51	42
What is an epigenetic change?	72	68	78	62	66	66	70	74	72
The DNA sequence in two different cells of one person, is how similar?	74	72	79	70	66	38	79	79	72
On average, how much of the variable DNA is the same in siblings?	31	31	33	21	34	71	31	30	32
Approximately how many genes does the human DNA code contain?	45	40	50	44	44	24	47	47	45
Genetic contribution to the risk for developing Schizophrenia comes from one gene or many genes?	67	59	77	52	62	58	66	73	64
What are polymorphisms?	75	70	81	62	68	59	76	81	72
‘Non-coding’ DNA describes DNA that does what?	78	70	83	62	71	74	75	83	74
What is genetic modification?	65	59	69	64	56	53	66	68	62
Can we predict a person’s behaviour from looking at their DNA sequence?	63	61	66	65	70	43	68	64	63
Is it true that in many countries, newborn infants are tested for certain genetic traits?	83	85	82	84	75	85	83	80	84
Genetic contribution to the risk for developing Autism comes from one or many genes?	68	61	74	57	72	64	72	70	67
Total	66	61	72	58	64	60	67	70	64

Numbers of participant for some groups do not add up to the total sample size due to missing data. For example, Men (1919) and Women (3301) do not sum to the total sample size because some participants opted for ‘non-binary’ or ‘prefer not to say’. The questions are short versions of the actual questions, retaining the essence but not the wording. Some questions have been rephrased here as their meaning was only clear in the context of the provided answers (see https://goldpsych.eu.qualtrics.com/jfe/form/SV_9zOfCcGhht7qwy9 for actual items)

The knowledge section also asked participants to rate how heritable, on a scale from 0 (no genetic influence) to 100 (entirely genetically influenced), the following traits were: height, weight, IQ, eye colour, clinical depression, motivation, school achievement and sexual orientation.

The attitudes section of iGLAS included 14 items asking participants about their views on various aspects of genetics. This section also included two vignettes to evaluate how people think genetic information can and should be used in evaluating people’s behaviour. In this paper, we present data for six of these items.

Demographic questions allowed stratified analyses by the following characteristics: *sex*, *education level*, *employment*, *parental status* (number of children), *country of secondary education*, *country of residence*, *religious affiliation*, *religiosity level*, spirituality level, *political ideology*, social media use, self-improvement and sources of guidance (e.g. counselling, self-help literature, religious guidance, consulting a psychic). In this paper, we present data for eight of the items (presented in italics above).

iGLAS was developed in English and Russian, with several phases of piloting and validation. It is also currently available in Romanian and French, with further translations planned. Data collection took place in both English and Russian internationally. Data used in this paper was collected using Qualtrics software (Qualtrics, Provo, UT).

### Participants

The total sample was 5405 participants: 845 (417 females; age M = 32.51, SD = 12.8) completed the English language version of iGLAS and 4559 (2887 females; age M = 30.43, SD = 8.0) completed the Russian language version. Participants had to be 18 or older, with no upper age limit. The English and Russian language samples were comparable in terms of age (English M = 32.51, SD = 12.77; Russian M = 30.43, SD = 8.00), sex and education level. The data reported here were collected between 31 October 2016 and 1 February 2017. The number of participants varied across different analyses due to missing data, as not all participants answered all questions. Despite the large sample, participants were not fully representatives of the countries in which they reside/received their secondary education: iGLAS was disseminated online, and so all participants were computer literate and had access to the internet; 88% of all respondents indicated that they had completed or were working towards university degree-level qualifications, within the context of the country in which they received their education.

Participants were reached through social media (https://facebook.com and https://vk.com), Reddit AMA (https://www.reddit.com/r/AMA/) and by emailing teachers in the UK via school circulars. A subsample included 112 undergraduate Psychology students from the University of London.

Participants from the USA were primarily recruited through an online science forum (Reddit Science AMA), and so respondents might reasonably be expected to have greater GK based on their engagement with such forums. However, analysis revealed no significant differences in GK between US-educated participants, whether they were recruited through the science AMA forum or not (*t* = 1.059, *p* = .291). In addition, participants from the USA were similar to participants outside the USA on level of education (*t* (5215) = −.289, *p* = .773) and religiosity (*t* (3995) = −.022, *p* = .983).

Informed consent was implemented at the beginning of the survey. The study was approved by the Goldsmiths Department of Psychology Ethics Committee and the Ethics Committee for Interdisciplinary Research of Tomsk State University, Russia.

## Results

Results showed unexpected sex differences, with men on average scoring higher on GK (M = 12.30, SD = 3.07, range 3–18) than women (M = 11.23, SD = 3.15, range 2–18), *t* (5218) = 11.84, *p* < 0.001 (Cohen’s *d* = 0.34). These differences were not explained by age or education. Data for men and women were normally distributed and covered almost the entire range of scores (see Table 1). For all inferential analyses, sex was regressed out.

- Hypothesis 1: Overall knowledge.

The GK section of the iGLAS questionnaire presented each question with 1 correct option and either 1 or 3 incorrect responses (see https://goldpsych.eu.qualtrics.com/jfe/form/SV_9zOfCcGhht7qwy9). The average GK was 11.8 (SD = 3.13, range 2–18), translating to an average correct score of 65.5%. Only 1.2% of participants got all the knowledge questions correct, and 3% of people achieved at or below the chance level of five correct answers.

Evaluation of individual items revealed some interesting gaps in knowledge (see Table 1). For example, less than 50% of participants knew the approximate number of genes in the human DNA, or the degree of genetic relatedness between family members. Approximately 30% of participants considered complex conditions, such as autism and schizophrenia, to be a product of a single genetic variant.

- Hypothesis 2: Estimates of Heritability.

Participants indicated the strength of genetic effects on eight common traits, using a sliding scale from 0 (not heritable) to 100 (entirely genetically determined).

**Fig. 1:**
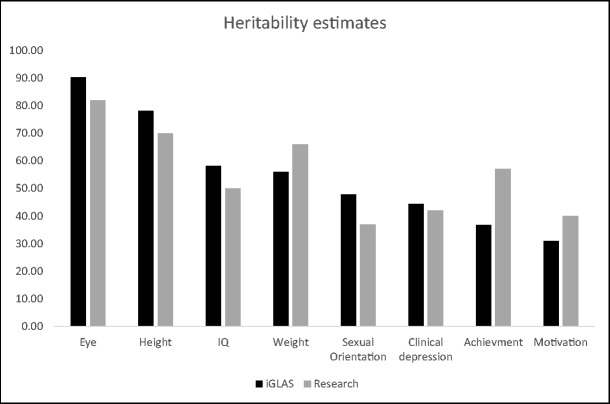
Average heritability as estimated by iGLAS participants vs. heritability from reputable genetic studies. iGLAS *N* ranged from 4803 to 5234 for different traits; the estimates came from the following sources: eye colour (Larsson et al. 2011); height (Jelenkovic et al. 2016); weight (Liu et al. 2015); school achievement (Rimfeld et al. 2015); IQ (Kovas et al. 2013); clinical depression (Lohoff 2010); motivation (Kovas et al. 2015); sexual orientation (LeVay 2016)

As presented in Fig. 1, participants’ estimates were close to the estimates established by behavioural genetic research. The pattern of under- and over-estimations was not random and confirmed our prediction: people tended to underestimate the heritability of weight, motivation and school achievement, but overestimate heritability of intelligence, height, eye colour and sexual orientation.

- Hypothesis 3: Cross-country differences

Participants received their secondary schooling in one of the 78 different countries, with 71.4% of the sample (3731 people) educated in Russia, 7.2% (375) in the UK, 6.1% (317) in Ukraine and 4.9% (255) in the USA. These four countries were represented in sufficient numbers for statistically meaningful comparisons. Levene’s test indicated equal variances across country groups (*F* = 2.14, *p* = .093). The results of the ANOVA revealed significant differences in the level of knowledge between the four countries: *F* (3, 4630) = 37.06, *p* < .001. However, the differences were small, with country of secondary education explaining only 2.3% of the variance in GK (eta sq = 0.023). Post hoc analysis revealed that people who received their secondary schooling in the USA (M = 13.66, SD = 2.84) scored on average significantly higher than participants educated in the other three countries; and that those educated in Ukraine (M = 11.11, SD = 3.26) scored significantly lower than those educated in Russia (M = 11.57, SD = 3.1). Participants educated in the UK (M = 11.22, SD = 3.17) differed significantly only from the US participants.

A similar pattern emerged when considering the country of residence. Three thousand five hundred ten participants were residents in Russia, 434 in the UK, 312 in the USA and 235 in Ukraine. Levene’s test indicated equal variances across country groups (*F* = 1.89, *p* = .129). The results of the ANOVA revealed significant differences in the level of knowledge between the four countries: *F* (3, 4487) = 40.06, *p* < .001. In this instance, the country of residence explained 2.6% of the variance in GK (eta sq = 0.026). Post hoc analysis revealed that current residents of the USA (M = 13.43, SD = 2.95) scored on average significantly higher than residents in the other three countries. Participants resident in Ukraine (M = 11.11, SD = 3.23), Russia (M = 11.51, SD = 3.1) and the UK (M = 11.35, SD = 3.19) each differed significantly from the USA, but not from each other.

- Hypothesis 4: Occupation

**Fig. 2:**
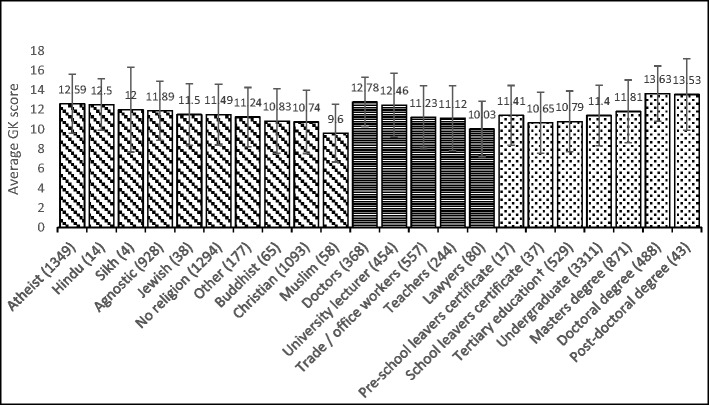
Mean GK scores for each group (number of participants in brackets) represented in the iGLAS study; †Tertiary education here refers to a level of study between the completion of compulsory schooling (school leavers’ certificates) and undergraduate studies. This is not applicable for all countries, but in the UK, tertiary education is referred to as further education, and is known as continuing education in the USA. Such education may be academic, practical and vocational or combinations of the three

Five professions/occupations were reasonably represented in our sample (see horizontally striped bars in Fig. 2) and were included for analysis. Doctors and lawyers both had smaller variance than the other three groups: ranges were 5–18 for doctors, 5–17 for lawyers, 3–18 for university lecturers, 2–18 for office workers and 3–18 for teachers. The lawyers’ data showed negative skew and the doctors’ data showed positive skew. Levene’s test indicated unequal variances (*F* = 8.89, *p* < .001). Therefore, Welch’s ANOVA was conducted (Field 2013) and revealed significant differences in the level of knowledge among the five professions: *F* (4, 436) = 32.43, *p* < .001. However, the differences were small, with occupation explaining 6.6% of the variance in GK (eta sq = 0.066). Post hoc analyses revealed that doctors and university lecturers had similar scores, but all other groups differed significantly from each other (Fig. 2).

- Hypothesis 5: Parental status

No significant differences in GK were found (*F* (1, 5283) = 0.32, *p* = .571) between participants identifying as having children (parents) (M = 11.55, SD = 3.21) and the rest of the participants (non-parents) (M = 11.66, SD = 3.14).

- Hypothesis 6. Educational levels

As expected, there was a significant positive correlation between education level and GK, *r* = .210, *n* = 5310, *p* < .001 (see Fig. 2). It is anticipated that this correlation would be stronger in the population as participants who had studied to at least degree level were over represented in this study. Additionally, the measure of education level used did not include type or level of qualification (e.g. degree class).

- Hypothesis 7: Religion

Participants identified their religious affiliation (see diagonal striped bars in Fig. 2) and rated their religiosity on a scale from 0 to 100, with 0 being a complete absence of religiosity. ‘Agnostic’, ‘Atheist’, ‘No religion’, and ‘Christian’ groups were represented in sufficient numbers for statistically meaningful comparisons.

Levene’s test revealed heterogeneity of variance for the four groups (*p* = .021). A one-way Welch ANOVA revealed that there was a significant difference in GK across the four groups, *F* (3, 2491.74) = 60.12, *p* < .001. However, the differences were small, with faith explaining 3.7% of the variance in GK (eta sq = 0.037). Games-Howell post hoc analyses revealed that each of the four groups differed significantly from the others (*p* < .05), with the biggest mean difference (*p* < .001) between Christians (M = 10.74, SD = 3.22, range 3–18) and Atheists (M = 12.59, SD = 3.02, range 2–18).

Participants were then grouped into either ‘believers’ (Christian, Buddhist, Hindu, Jewish, Muslim, Sikh and others; *N* = 1402) or ‘non-believers’ (Agnostic, Atheist and no religion; *N* = 3492). Levene’s test revealed heterogeneity of variance for the two groups (*p* = .029). A one-way Welch ANOVA revealed that there was a significant difference in GK between believers (M = 10.80, SD = 3.20, range 3–18) and non-believers (M = 12.01, SD = 3.08, range 2–18); *F* (1, 2601.93) = 124.193, *p* < .001. However, the differences were small, with belief explaining 2.5% of the variance in GK (eta sq = .025). This indicates that not all religious and non-religious groups show the same pattern of GK, with some religious groups likely out performing non-religious groups, as can be seen in Fig. 2.

For the entire sample, religiosity (measured on a scale of 0 to 100) was normally distributed (skew = 1.2, kurtosis .581). There was a significant weak negative correlation between religiosity and GK, *r* = −.124, *n* = 4297, *p* < .001. Additional analyses were performed within the religious groups. Religiosity was normally distributed within the Christian group (skew = −.015, kurtosis = .695) and was not correlated with GK (*r* = −.007, *n* = 1079, *p* = .825). Therefore, poorer GK is associated with self-identifying as Christian, not the level of one’s religiosity (devotion to Christianity). A similar intra-faith correlation pattern was seen for Muslim (*r* = .040, *n* = 58, *p* = .766) and Buddhist (*r* = −.113, *n* = 65, *p* = .370) participants. This may indicate that there is a very weak association between religiosity and GK which is only identifiable from the large size of the entire sample.

- Hypothesis 8: Political views

Participants rated their position on a political spectrum from 0 (left/very liberal) to 10 (right/very conservative). Although the concept of a left/right political spectrum is less applicable to the Eastern European concept of political affiliation (Đorić and Filipović 2010), the concept of liberal vs. conservative is comparable for the English- and Russian-speaking participants. On this spectrum, 49.9% of participants identified as liberal (answering 0–4), 26.7% as centre (answering 5) and 23.4% as conservative (answering 6–10). Significant numbers of participants (17.3 and 5.9%) identified as extremely liberal (scoring 0–2) and extremely conservative (scoring 8–10), respectively. There was a weak but significant negative correlation between political ideology and GK: those to the left of the political spectrum had slightly better GK than those to the right *r* = −.053, *n* = 3861, *p* < .001.

- Hypothesis 9: Genetic knowledge and determinism

We assessed whether higher GK is associated with less deterministic views of genetics, by correlating GK with two ‘determinism’ questions: ‘I believe that my destiny is written in my genes’ and ‘If genes influence our behaviour then there is no free will’. These two questions were measured on a 7-point Likert scale (strongly disagree–strongly agree). Visual inspection showed that both questions were positively skewed (though skewness was less than 2 in both instances). Most of the participants (70.7%) disagreed that their destiny is written in their genes, 25.5% reported that they agree and 3.7% neither agreed nor disagreed. For the item ‘If genes influence our behaviour then there is no free will’, 85.5% of participants disagreed, 9% agreed to some extent and 5.5% neither agreed nor disagreed.

A weak negative correlation *r* = −.052, *n* = 5301, *p* < .001 was found between GK and the ‘belief that one’s destiny is written in one’s genes’. Similarly, a weak negative correlation *r* = −.120, *n* = 5295, *p* < .001 was found between GK and ‘belief that genetic influences result in there being no free will’. As this question is negatively phrased, this indicates that greater GK was associated with less deterministic views.

- Hypothesis 10: Genetic knowledge and genetic testing

iGLAS included four items about willingness to undergo genetic testing: ‘If genetic testing allowed you to have improved treatment (for example, medication with fewer side effects) how likely would you be to take that test?’ measured on a 7-point Likert scale (strongly disagree–strongly agree); ‘In each of the scenarios below, please indicate how likely you would be to take up the offer to have your genome sequenced?: If there were (no/moderate/definite) history of debilitating disease in your family.’ In this question, each participant was asked to respond based on each of the scenarios in parentheses above. This question was measured on a 100-point slider scale (not at all likely to very likely).

Most participants (88.6%) expressed willingness to undergo genetic testing if it were to improve their treatment. In the condition of high familial risk (disorder/illness running in the family), 43% of participants expressed extreme likelihood to undergo personal genetic testing; the percentages were 33.7 and 23.7 in the medium and low familial risk conditions, respectively.

There was a weak, but statistically significant, positive correlation between GK and each of the four items (*p* < .001). Higher GK was associated with greater willingness to undergo testing *r* = .208 (*N* = 5304) for improved treatment, *r* = .229 (*N* = 4709) for low familial risk, *r* = .253 (*N* = 4859) for moderate familial risk, and *r* = .219 (*N* = 4830) for high familial risk.

iGLAS also included two items tapping into trust in research institutions and suspicion about genetic research. Trust and suspicion were measured with 2 items: ‘I do not trust research institutions in my country because they might misuse the data obtained from participants’ measured on a 7-point Likert scale (strongly disagree—strongly agree); and ‘I feel suspicious about genetic studies; hidden political/economic agendas may be behind them.’ measured on a 7-point Likert scale (strongly disagree–strongly agree). Some degree of suspicion (i.e. above neutral on the testing scale) towards genetic studies was reported by 19.1% of people, and 12.3% of participants reported a lack of trust in the research institutions in their country by responding below neutral.

A simple linear regression was conducted to examine whether willingness to undergo genetic testing for improved treatment was associated with GK, trust in research institutions and suspicion about genetic research. The model explained a small but significant proportion of variance in willingness to undergo testing, *R*2 = .079, *F* (3, 5282) = 151.121, *p* < .001. GK and suspicion of genetic studies significantly predicted willingness to undergo genetic testing (*B* = .175, *t* (5282) = 13.03, *p* < .001; *B* = −.192, *t* (5282) = − 13.19, *p* < .001, respectively). Trust in research institutions did not significantly predict willingness to undergo genetic testing scores (*B* = −.002, *t* (5282) = −.168, *p* = .886).

## Discussion

This study used the International Genetic Literacy and Attitude Survey (iGLAS; Chapman et al. in press) to assess genetic knowledge and attitudes of 5404 participants from diverse backgrounds. The average score on basic genetic knowledge was 11.62 out of 18 (65.5%). This indicates poor genetic literacy, considering the multiple-choice format which significantly increases the chances of correct responses, even from people with minimal knowledge (Wilkinson and Shaw 2015). Therefore, scores close to 100% correct are expected from genetically literate people. Furthermore, 87.6% of our respondents were educated to degree level or higher—a significantly greater proportion than in the represented populations. For example, as of 2015, 38.7% of European citizens had studied until at least degree level, up by 15.1% from 2002 (Eurostat 2015). For the USA, 33.5% of 25 to 29 years old hold at least a Bachelor’s degree (2013), an increase of 8.8% from 1995 (Rampell 2013). It is therefore likely that population average GK is even lower than found in this study. This is concerning as the questions included genetic concepts that are fundamental for understanding how genes affect our lives.

The results suggested a positive correlation between education level and GK. As our sample is skewed towards higher education, it is reasonable to expect an even stronger correlation between education and knowledge in the general population. However, with increasing numbers of school leavers (those who complete compulsory education) attending university, factors other than educational attainment (years in education) may contribute to GK: the quality of education, educational achievement, the types of degree and if/when/how genetics was included in the school curriculum.

The study also revealed specific gaps in genetic knowledge. For example, ~ 30% of participants thought that schizophrenia and autism were the product of a single genetic mutation, when in fact research has consistently shown that they stem from multiple genetic factors (Bergen and Petryshen 2012; van Eijk et al. 2015), which also interact with environments. Discovery of the polygenic nature of most human traits, including all common diseases and disorders, is of great importance. The shift towards understanding that traits are polygenic (and not caused by a single mutation) represents a fundamental qualitative change in the way a person views genetic effects and the traits themselves. The significance of this change can be likened to the tremendous mental changes occurring with the shift from being illiterate to literate (D’Angelo 1982).

Overall, participants provided reasonably accurate estimates of heritability—the extent to which genetic factors contribute to individual differences in traits. However, people on average underestimated genetic influences on weight, motivation, and school achievement. In contrast, they overestimated heritability of eye colour, height, sexual orientation, and IQ. This pattern of under- and over-estimation is likely driven by an erroneous intuition that certain traits are more easily controllable or malleable than others, and therefore are under weaker genetic control. A powerful example of this is the common belief that educational achievement is less heritable than IQ, as evidenced in this study. Research, however, has shown that for school children, heritability is greater for academic achievement than for intelligence (Kovas et al. 2015; Krapohl et al. 2014).

The analyses stratified by different demographic characteristics revealed several interesting findings. Lack of knowledge and misconceptions were evident across all occupation groups, including medical doctors, teachers, lawyers, university lecturers, and office workers. This lack of knowledge raises cause for concern because of the importance of genetic awareness for the roles these professions play: teachers and lecturers—in education; medical doctors—in health and wellbeing; and lawyers—in legal representation and reform. Office workers were included to provide a control sample, but their results also highlight weaknesses in genetic knowledge in general.

The relatively low level of genetic knowledge was also evident across all belief groups, with slightly lower average scores for individuals who were identified as religious than those who were identified as non-religious. Contrary to our hypothesis, no differences were found in GK between parents and non-parents, and those who were identified as more conservative had on average poorer genetic knowledge than those more liberal.

With regard to attitude towards genetics, this study identified that 88.6% of participants would consider undergoing genetic testing to access improved health care. This is in line with a previous study, in which 85% of participants responded positively towards a question about their own willingness to undergo predictive genetic testing for preventable health conditions (Makeeva et al. 2010).

The results also suggest that people with greater genetic knowledge are more likely to benefit from genetic advances, such as greater willingness to opt for genetic testing for medical reasons. As suggested by the negative correlation between GK and determinism, people with greater GK are likely to have a more realistic view on the sources of individual differences. However, the correlation was weak, indicating that many factors beyond knowledge influence genetic-related deterministic views.

The results of the study also indicate that GK of a population may depend on such factors as curricula, policy, legislation, and the media. For example, the observed higher rates of GK for those resident and/or educated in the USA may have resulted from specific cultural contexts, such as direct to consumer genetic testing products and advertisements. Certain high-profile media cases such as Angelina Jolie’s decision to undergo BRCA genetic testing and prophylactic mastectomy (Jolie 2013) may have also increased public awareness and interest in genetics. Availability of genetic testing and genetic counselling through health service providers may also explain some of the cross-cultural differences observed in this study. For example, Henneman et al. (2004) identified that higher levels of genetic knowledge were associated with familiarity with genetic testing.

To achieve genetic literacy, societies can take several immediate steps. Genetics should be included as part of the curriculum for training teachers, doctors and nurses, psychologists and other professionals. Including an up-to-date genetic curriculum in this key professionals’ training will have a cascading effect, with knowledge reaching children, parents and society at large. All stakeholders, including research institutions, funding bodies, and media companies, should aim to provide clear and accurate genetic information to the public. Individuals willing to improve their genetic knowledge can use the freely available excellent tools and resources, including the Genetic Science Learning Centre (http://learn.genetics.utah.edu/), Centre for Genetics Education (http://www.genetics.edu.au/), the National Institutes of Health (https://www.genome.gov/27527634/competency-and-curricular-resources/) and yourgenome.org. Achieving universal genetic awareness and literacy also requires government-led efforts. These steps will bring immediate and long-term benefits to individuals and society, not least of all through improved engagement with genomic-led medical and health reforms.
